# Screening of mRNA Chemical Modification to Maximize Protein Expression with Reduced Immunogenicity

**DOI:** 10.3390/pharmaceutics7030137

**Published:** 2015-07-23

**Authors:** Satoshi Uchida, Kazunori Kataoka, Keiji Itaka

**Affiliations:** 1 Laboratory of Clinical Biotechnology, Center for Disease Biology and Integrative Medicine, Graduate School of Medicine, The University of Tokyo, 7-3-1 Hongo, Bunkyo-ku, Tokyo 113-0033, Japan; E-Mail: suchida@bmw.t.u-tokyo.ac.jp; 2 Department of Bioengineering, Graduate School of Engineering, The University of Tokyo, 7-3-1 Hongo, Bunkyo-ku, Tokyo 113-0033, Japan; 3 Department of Materials Engineering, Graduate School of Engineering, The University of Tokyo, 7-3-1 Hongo, Bunkyo-ku, Tokyo 113-0033, Japan

**Keywords:** mRNA, modification, immunogenicity, translation efficiency, nuclease-mediated degradation

## Abstract

Chemical modification of nucleosides in mRNA is an important technology to regulate the immunogenicity of mRNA. In this study, various previously reported mRNA formulations were evaluated by analyzing *in vitro* protein expression and immunogenicity in multiple cell lines. For the macrophage-derived cell line, RAW 264.7, modified mRNA tended to have reduced immunogenicity and increased protein expression compared to the unmodified mRNA. In contrast, in some cell types, such as hepatocellular carcinoma cells (HuH-7) and mouse embryonic fibroblasts (MEFs), protein expression was decreased by mRNA modification. Further analyses revealed that mRNA modifications decreased translation efficiency but increased nuclease stability. Thus, mRNA modification is likely to exert both positive and negative effects on the efficiency of protein expression in transfected cells and optimal mRNA formulation should be determined based on target cell types and transfection purposes.

## 1. Introduction

Therapeutic delivery of messenger RNA (mRNA) is a new method of providing therapeutic proteins and peptides for cells [1,2]. It has several advantages over DNA delivery: mRNA delivery lacks the risk of insertion mutagenesis after random genomic integration, and can be efficiently transfected into non-dividing cells without the need for nuclear entry. When compared to the delivery of proteins and peptides, mRNA delivery had a prolonged effect. mRNA delivery can also be used to express proteins and peptides that would work in the intracellular space, such as transcription factors, whereas such proteins are difficult to deliver directly through the cell membrane. mRNA delivery can be used to generate induced pluripotent stem cells (iPSCs) in a safe manner without genomic integration of the delivered gene [3,4]. mRNA delivery is also a safe option for introducing programmable nucleases, such as zinc finger nuclease (ZFN), TAL effector nuclease (TALEN), and CRISPR/Cas9, for genome editing [5,6,7]. In addition to these examples of *in vitro* delivery, we and other groups have shown the feasibility of *in vivo* mRNA delivery for treating disease in animal models, such as surfactant B deficiency, cancer, myocardial infarction, and sensory nerve dysfunction [8,9,10,11].

One of the major obstacles to therapeutic application of mRNA delivery is the high immunogenicity of mRNA molecules [12,13]. mRNA is recognized by pattern recognition receptors (PRRs), such as Toll-like receptor 3, 7, 8, and retinoic acid-inducible gene I (RIG-I), resulting in a strong inflammatory response. To date, this has limited the application of mRNA mainly to vaccine therapy [14,15,16]. Minimizing the immunogenicity of mRNA will expand the therapeutic application of mRNA delivery. Chemical modification of mRNA is a promising technology to reduce immunogenicity by preventing recognition of mRNA by PRRs [4,8,17,18].

Several groups have contributed to the development of mRNA modification, but there were differences in modification protocols between the groups probably because they used different target cells and animal models [4,8,17,18]. Considering future *in vivo* studies and clinical applications, it is worth screening the formulations of modified mRNA depending on the applications. In this study, we evaluated various reported formulations for protein translation efficiency, immunogenicity, and nuclease-mediated degradation using various cell types [3,8,19,20].

## 2. Experimental Section

### 2.1. Preparation of mRNA

mRNA was prepared following the protocol described previously with slight modification [4]. mRNA was transcribed *in vitro* from plasmid DNA (pDNA) templates using a MEGAscript T7 Transcription Kit (Ambion, Austin, TX, USA). For construction of the template pDNA, protein-expressing fragments were inserted into the pSP73 vector under expression control from a T7 promoter. The protein-expressing fragments included *Gaussia* luciferase (GLuc) derived from the pCMV-GLuc control plasmid (New England BioLabs, Ipswich, MA, USA) and a synthetic firefly luciferase (Luc2) from pGL4.13 (Promega, Madison, WI, USA). A 120-bp poly A/T sequence was cloned into the pSP73 vector to attach a poly(A) chain to the 3′ end of the mRNA. Before *in vitro* transcription (IVT), the plasmids were linearized downstream of the poly(A) tail. For mRNA modification, chemically modified nucleosides, such as 5-methyl-cytidine (5mC), 2-thio-uridine (2sU), and pseudo-uridine (ψU), were purchased from TriLink (San Diego, CA, USA) and added to the IVT reaction solution at the indicated ratios to substitute for cytidine (5mC) and uridine (2sU and ψU). For 5′ capping of mRNA, an anti-reverse cap analog (ARCA, 3′-*O*-Me-m7G(5′)ppp(5′)G RNA Cap, New England BioLabs) was added to the IVT solution. Transcribed mRNA was purified with a MEGAclear kit (Ambion), and the concentration was determined by absorbance at 260 nm.

### 2.2. Cells

Raw 264.7 and HuH-7 cells were obtained from the Riken Cell Bank (Tsukuba, Japan). Human mesenchymal stem cells (MSCs) were purchased from Lonza (Allendale, NJ, USA) and used for the experiments at passage 5. Mouse embryonic fibroblasts were isolated from embryos (E12.5) of ICR mice (Charles River Laboratories, Yokohama, Japan). After removing head and visceral tissues from the embryo, the remaining tissues were minced using scissors. To prepare cell suspensions, the tissue was incubated in a stirred solution of 0.05% trypsin and 0.5 mM EDTA at room temperature for 30 min. The suspension was filtered through 70-μm nylon mesh. All animal protocols were conducted with the approval of the Animal Care and Use Committee (P11-076, 16 January 2012), The University of Tokyo, Tokyo, Japan.

All cell types were cultured in Dulbecco’s Modified Eagle’s Medium (DMEM) containing 10% fetal bovine albumin (FBS, HyClone Laboratory, GE Healthcare Life Science, South Logan, UT, USA) and 1% penicillin/streptomycin (Sigma–Aldrich, St. Louis, MO, USA).

### 2.3. mRNA Introduction to Raw 264.7 Cells

Raw 264.7 cells were seeded onto six-well plates at a density of 800,000 cells/well. After 24 h of incubation, the culture medium was replaced with serum-free Opti-MEM medium (Invitrogen, Carlsbad, CA, USA), and mRNA encoding GLuc was added to each well using Lipofectamine™ LTX (Invitrogen), a commonly used cationic lipid-based transfection reagent, following the manufacturer’s protocol. In brief, for each well, 2.5 μL of PLUS™ solution was added to 2.5 μg of mRNA dissolved in 250 μL of Opti-MEM medium, and the resulting solution was mixed with 250 μL of Opti-MEM medium containing 6.25 μL of Lipofectamine™ LTX reagent. After 4 h of transfection, cells were harvested for extraction of total mRNA and the culture medium was collected to evaluate GLuc expression. In addition, cell viability was measured using Cell Counting Kit-8 (Dojindo, Kumamoto, Japan), following the manufactures’ protocol.

Total RNA was extracted using an RNeasy Mini kit (Qiagen, Hilden, Germany) and reverse-transcribed to complementary DNA (cDNA) using ReverTra Ace^®^ qPCR RT Master Mix kit (Toyobo Life Science, Osaka, Japan). Quantitative real-time PCR (qRT-PCR) was performed using an ABI Prism 7500 Sequence Detector (Applied Biosystems, Foster City, CA, USA) and TaqMan^®^ Gene Expression Assays (Applied Biosystems). Mm00439552_s1 was used for interferon-β1, Mm00446190_m1 was used for interleukin 6, Mm00443258_m1 was used for tumor necrosis factor α, and Mm00434228_m1 was used for interleukin 1β. Gene copy numbers were standardized to β-actin (Taqman^®^ Gene Expression Assay, Mm00607939).

The level of GLuc expression in medium was measured with *Renilla* Luciferase Assay System (Promega) and the GloMaxTM 96 microplate luminometer (Promega).

### 2.4. mRNA Transfection into HuH-7, MEFs and MSCs

HuH-7, MEFs, and MSCs were seeded onto 96-well plates at a density of 5000 cells/well. After 24 h of incubation, media were replaced with DMEM containing 10% FBS, and 0.1 μg of mRNA encoding GLuc or Luc2 was added to each well using Lipofectamine™ LTX (Invitrogen) or linear polyethyleneimine (LPEI, JetPEI, Polyplus transfection™, Illkirch, France), a commonly used polycation-based transfection reagent. Lipofectamie™ LTX/mRNA complex was prepared at the same mixture ratio as that in the transfection to Raw 264.7 cells (0.1 μg of mRNA was mixed with 0.25 μL of Lipofectamine™ LTX reagent, and 0.1 μL of PLUS™ solution for each well), and LPEI/mRNA complex was prepared at an N/P (amines in LPEI/phosphates in mRNA) ratio of 8. After 24 h of transfection, GLuc expression in the medium was quantified as described in the previous section and Luc2 expression in the cell lysate was quantified using a Luciferase Assay system (Promega).

### 2.5. Cell-Free Translation

Translational efficiency of mRNA was evaluated using a cell-free translational system, Rabbit Reticulocyte Lysate (Promega), following the manufacturer’s protocol. Briefly, 0.6 μg of mRNA encoding GLuc was incubated in 15 μL of rabbit reticulocyte lysate at 30 °C. After 90 min, GLuc expression in lysate was measured as described in Section 2.3.

### 2.6. Evaluation of Nuclease-Mediated Degradation

Naked mRNA at 25 ng/μL was incubated with 0.05% FBS for 15 min at 37 °C, and then purified using an RNeasy Mini kit. qRT-PCR was performed with an ABI Prism 7500 Sequence Detector to quantify the mRNA with an intact sequence between the primer pair. The primer pair (forward: TCTGTTTGCCCTGATCTGC, reverse: CCCTTGATCTTGTCCACCTG) amplifies 528 bp of GLuc mRNA. The amount of mRNA after serum incubation was compared to that before serum incubation and the relative values were reported.

## 3. Results

### 3.1. mRNA Transfection into Immune Cells

Six previously reported modified mRNA formulations were evaluated in this study [3,8,19,20]: (1) 10% 5mC, 10% 2sU; (2) 25% 5mC, 25% 2sU; (3) 20% 5mC, 10% 2sU, 10% ψU; (4) 100% 5mC; (5) 100% ψU; (6) 100% 5mC, 100% ψU. The percentages for each formulation indicate the percentage of total cytidine or uridine in mRNA that is a modified cytidine or uridine analog. These formulations will be abbreviated as (1) mC_10%_ sU_10%_, (2) mC_25%_ sU_25%_, (3) mC_20__%_ sU_10%_ ψU_10%_, (4) mC_100%_, (5) ψU_100%_ and (6) mC_100%_ ψU_100%_.

The immunogenicity of each formulation was evaluated by mRNA transfection into RAW264.7 cells (a mouse leukemic monocyte macrophage cell line). The modified mRNA encoded the reporter *Gaussia* luciferase (GLuc), a secretable form of luciferase [21]. In this experiment, we used serum-free medium with the intention to focus on the effect of mRNA immunogenicity by excluding the effect of the other factors such as degradation in the cultured medium. Four hours after mRNA transfection using Lipofectamine™ LTX, the expression of the inflammatory factors interferon-β (IFN-β), interleukin-6 (IL-6), tumor necrosis factor-α (TNF-α), and interleukin-1β (IL-1β) was measured by qRT-PCR.

**Figure 1:**
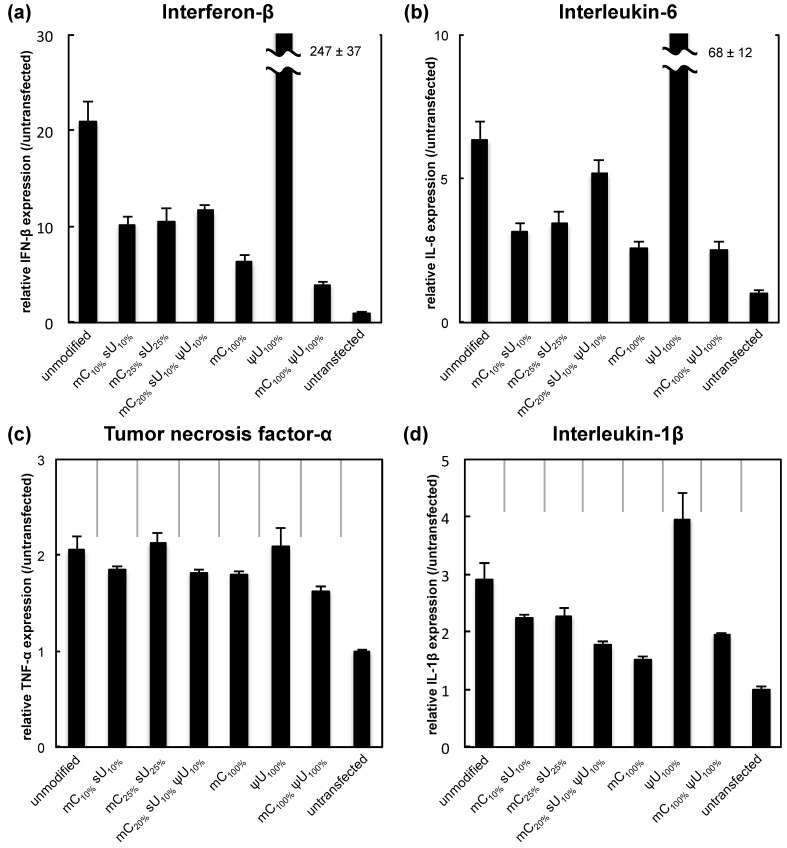
Inflammatory response after mRNA introduction into RAW264.7 cells. mRNA-encoding GLuc was introduced to RAW264.7 cells using Lipofectamine™ LTX. After 4 h, expression of inflammatory molecules was measured with qRT-PCR. (**a**) Interferon-β (IFN-β); (**b**) Interleukin-6 (IL-6); (**c**) Tumor necrosis factor-α (TNF-α); and (**d**) Interleukin-1β (IL-1β). Data are presented as the mean ± standard error of the mean (S.E.M.) (*N* = 6) for expression levels relative to the untransfected control.

The expression of IFN-β and IL-6 increased remarkably after transfection of unmodified mRNA compared with the untransfected control (Figure 1). For five of the modified mRNA formulations, except for ψU_100%,_ the expression showed only a mild increase from the untransfected control. Among the modification formulations, the mC_100%_ and the mC_100%_ ψU_100%_ formulations had the most reduced inflammatory responses. In addition, cell viability was evaluated at the same time point. While transfection of unmodified mRNA caused some decrease in cell viability, the use of modified mRNAs generally alleviated the cytotoxicity (Figure S1). By making scatter plots representing the correlation between cytokine induction and cell viability after mRNA transfection (Figure S2), it is well documented that the cell viability inversely correlated with the cytokine production with the exception of ψU_100%_.

**Figure 2:**
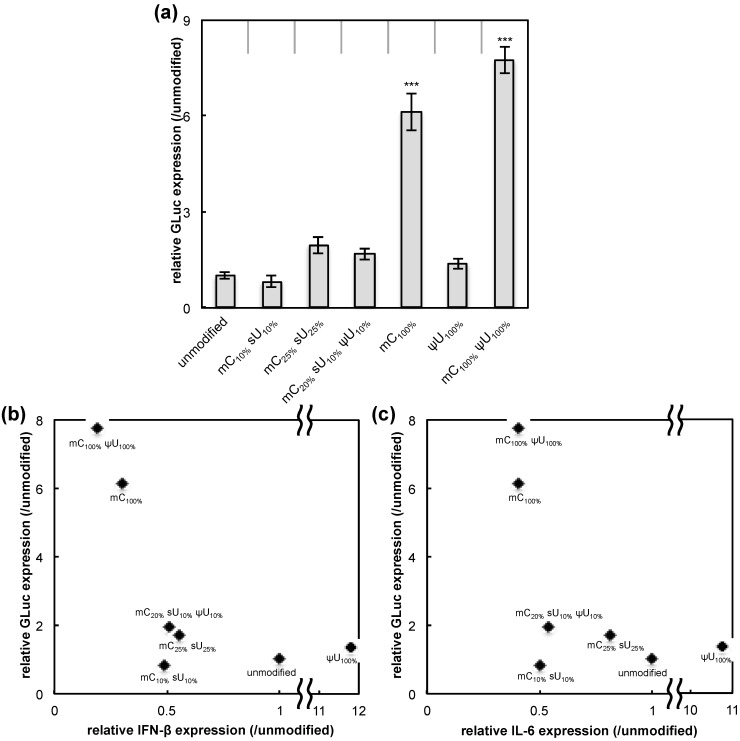
GLuc expression in RAW264.7 cells. mRNA-encoding GLuc was introduced to RAW264.7 cells using Lipofectamine™ LTX. After 4 h, the amount of GLuc protein in culture media was measured. (**a**) Efficiency of GLuc expression. The graph shows relative values compared to GLuc expression after transfection of unmodified mRNA. Data are presented as the mean ± standard error of the mean (S.E.M.) (*N* = 6). Statistical significance was assessed by one-way analysis of variance (ANOVA) followed by Dunnett’s test. *** *p* < 0.001 *versus* unmodified mRNA group. (**b,c**) Correlation between inflammatory responses and GLuc expression. The *x*-axis shows the expression of (**b**) interferon-β (IFN-β) and (**c**) interleukin-6 (IL-6) (see Figure 1). The *y*-axis showed the efficiency of GLuc expression (panel (**a**) of this figure).

The protein expression efficiency for each formulation was determined by measuring GLuc secreted into the culture media. The protein expression efficiency was then compared with the IFN-β and IL-6 response induced by each formulation. Four hours after transfection, GLuc expression was significantly increased for the mC_100%_ and the mC_100%_ ψU_100%_ formulations while the other four formulations had GLuc expression levels similar to that of the unmodified mRNA (Figure 2a). mRNA with mC_100%_ and mC_100%_ ψU_100%_ exhibited high transgene expression with less induction of an inflammatory response (IFN-β or IL-6) (Figure 2b,c). Of the formulations tested, these two are likely optimal for obtaining high protein yield with low immunogenicity. In contrast, the mC_10%_ sU_10%_, mC_25%_ sU_25%_, and mC_20%_ sU_10%_ ψU_10%_ formulations have reduced immunogenicity, but they did not show significant increase in luciferase expression compared to that of the unmodified mRNA. mRNA with ψU_100%_ resulted in GLuc expression comparable to unmodified mRNA, but it induced a much stronger inflammatory response. In accordance with these results, although cell viability was positively correlated with luciferase expression efficiency, some formulations, especially mC_25%_ sU_25%_, exhibited only a small increase in luciferase expression despite remarkable increase in cell viability (Figure S2). These results indicate that the reduced inflammatory responses, as well as the increase in cell viability, are not always correlated with an increase in protein expression efficiency, motivating us to analyze the effect of mRNA modification using other cell types.

### 3.2. Influence of Transfection Conditions on Protein Expression from mRNA

**Figure 3:**
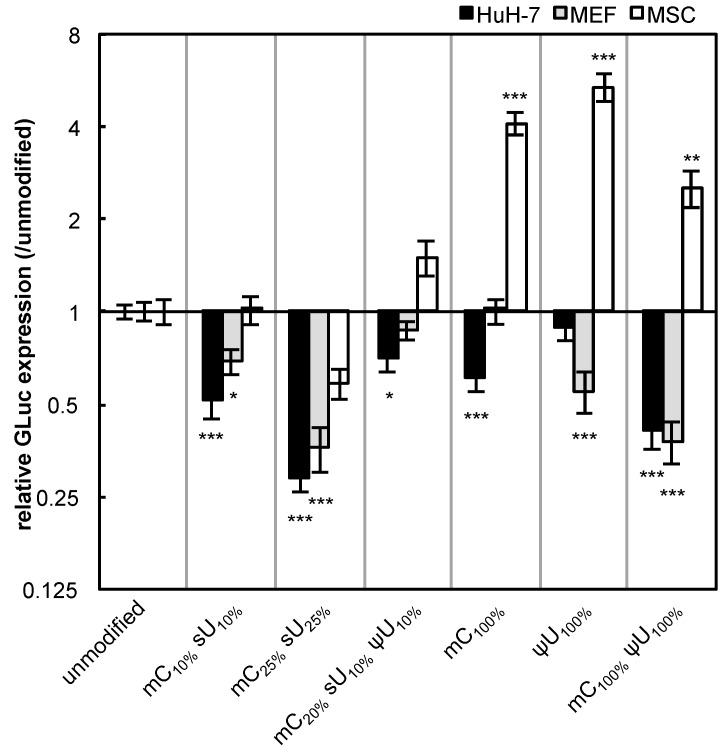
GLuc expression in HuH-7 cells, MEFs, and MSCs. mRNA-encoding GLuc was introduced to HuH-7 cells (black bars), MEFs (gray bars), and MSCs (white bars) using Lipofectamine™ LTX. After 24 h, the amount of GLuc protein in culture media was measured. The graph shows relative values compared to GLuc expression after transfection of unmodified mRNA. Data are presented as the mean ± standard error of the mean (S.E.M.) (*N* = 6). Statistical significance was assessed by one-way analysis of variance (ANOVA) followed by Dunnett’s test. * *p* < 0.05, ** *p* < 0.01, *** *p* < 0.001 *versus* unmodified mRNA.

The non-immune cells (cancer-derived cell line (HuH-7), primary cells (MEFs), and adult tissue stem cells (MSCs)) were used for evaluating the effect of mRNA modification on protein expression efficiency. In these experiments, we used serum-containing medium with the intention of evaluating the combinational effect of various factors that can affect *in vitro* transfection. Unexpectedly, most of the formulations had reduced GLuc expression in HuH-7 cells and MEFs after 24 h of transfection (Figure 3). In contrast, in MSCs, modified mRNA generally exhibited higher GLuc expression than the unmodified mRNA. The mC_100%_ and ψU_100%_ formulations had the highest GLuc expression in MSCs. Protein production efficiency from modified mRNA depends on cell type.

**Figure 4:**
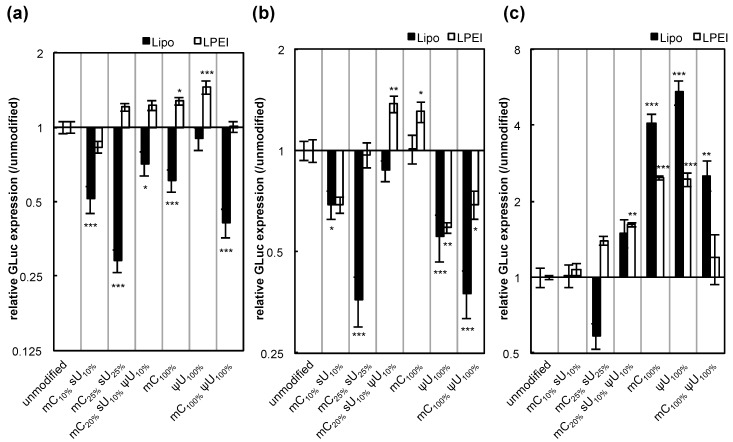
GLuc expression efficiency after mRNA introduction using two different transfection reagents. Two types of transfection reagents, Lipofectamine™ LTX (Lipo, black bars) and linear polyethyleneimine (LPEI, white bars), were used for mRNA introduction. mRNA-encoding GLuc was introduced to (**a**) HuH-7, (**b**) MEFs, and (**c**) MSCs. After 24 h, the amount of GLuc protein in culture media was measured. The graph shows relative values compared to GLuc expression after transfection of unmodified mRNA. For Lipo-treated groups, the same data shown in Figure 3 are presented. Data are presented as the mean ± standard error of the mean (S.E.M.) (*N* = 6). Statistical significance was assessed by one-way analysis of variance (ANOVA) followed by Dunnett’s test. * *p* < 0.05, ** *p* < 0.01, *** *p* < 0.001 versus unmodified mRNA.

**Figure 5:**
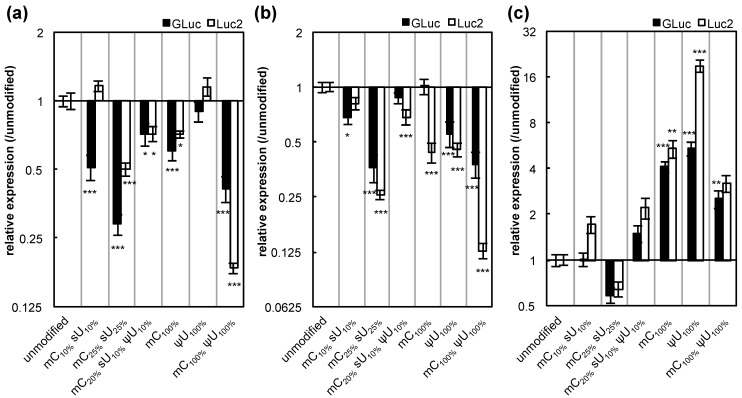
Influence of mRNA modification on the expression of GLuc and Luc2. Two genes, *Gaussia* luciferase (GLuc, black bars) and a synthetic firefly luciferase (Luc2, white bars), were introduced to (**a**) HuH-7 cells, (**b**) MEFs, and (**c**) MSCs using Lipofectamine™ LTX. After 24 h, GLuc protein in culture media and Luc2 protein in cell lysates were measured. The graph shows relative values compared to the expression after transfection of unmodified mRNA. For GLuc-treated cells, the data is the same as in Figure 3. Data are presented as the mean ± standard error of the mean (S.E.M.) (*N* = 6). Statistical significance was assessed by one-way analysis of variance (ANOVA) followed by Dunnett’s test. * *p* < 0.05, ** *p* < 0.01, *** *p* < 0.001 *versus* unmodified mRNA.

We then evaluated other factors that may affect protein expression efficiency, such as transfection reagents and the gene encoded by the mRNA. When linear polyethyleneimine (LPEI) was used for transfection, the efficiency with the control unmodified mRNA was comparable to that using Lipofectamine™ LTX (Figure S3). However, the effect of mRNA modification on the protein expression greatly varied with the transfection reagents, particularly in HuH-7 cells (Figure 4). It is speculated that these complex results may be due to the different intracellular mechanisms between the two reagents. Regarding genes encoded by the mRNA, the expression of firefly luciferase (Luc2) showed trends similar to GLuc (Figure 5). However, large differences between the two genes were observed in some cases.

### 3.3. Evaluation of Translation Efficiency and Nuclease Stability of Modified mRNA

Although the mRNA modification showed reduced immunogenicity in immune cells, it may cause negative effects on protein expression efficiency in non-immune cells (Figure 3, Figure 4 and Figure 5). To directly analyze protein translation, we used a cell-free translational system based on rabbit reticulocyte lysates containing all molecules that would be required for protein translation from mRNA. Using this system, translation efficiency can be measured independent of other factors such as cellular uptake, endosomal escape, and transport of mRNA to the place of translation.

**Figure 6:**
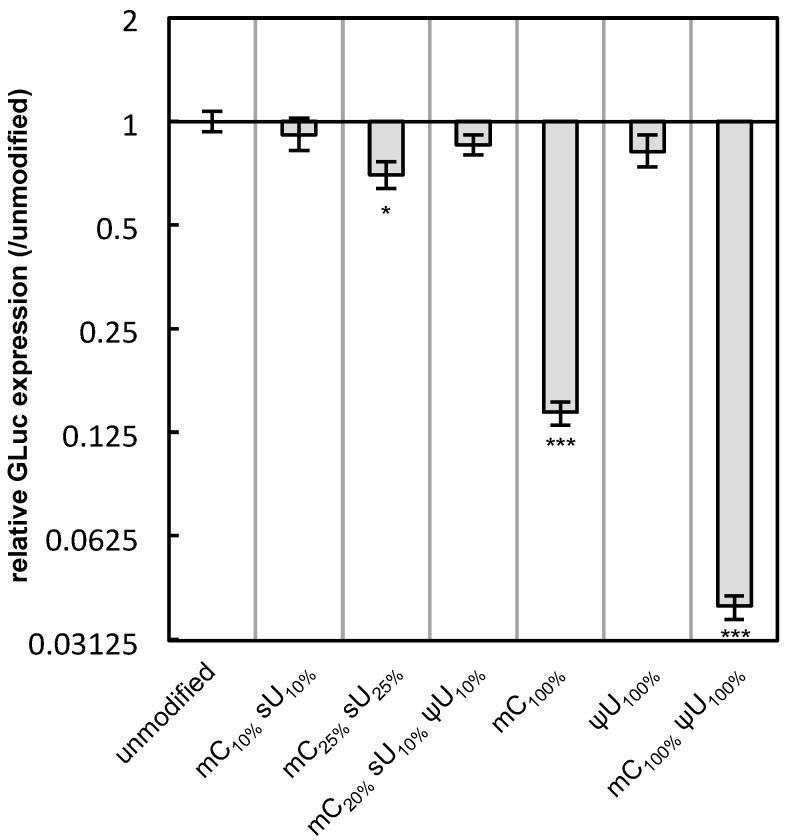
Translation efficiency of modified mRNA. mRNA-encoding GLuc in a naked form was incubated in a cell-free translation system composed of rabbit reticulocyte lysate. After 90 min, GLuc was measured. The graph shows relative values compared to the expression of unmodified mRNA. Data are presented as the mean ± standard error of the mean (S.E.M.) (*N* = 4). Statistical significance was assessed by one-way analysis of variance (ANOVA) followed by Dunnett’s test. * *p* < 0.05, *** *p* < 0.001 *versus* unmodified mRNA.

Translational activity tended to decrease after mRNA modification compared to that of unmodified mRNA, although the extent of the decrease was considerably different depending on the formulation (Figure 6). The mRNA with mC_100%_ and mC_100%_ ψU_100%_, which had increased protein expression in Raw 264.7 cells and MSCs (Figure 2, Figure 3, Figure 4 and Figure 5), showed a large decrease in protein translation in the cell-free system.

**Figure 7:**
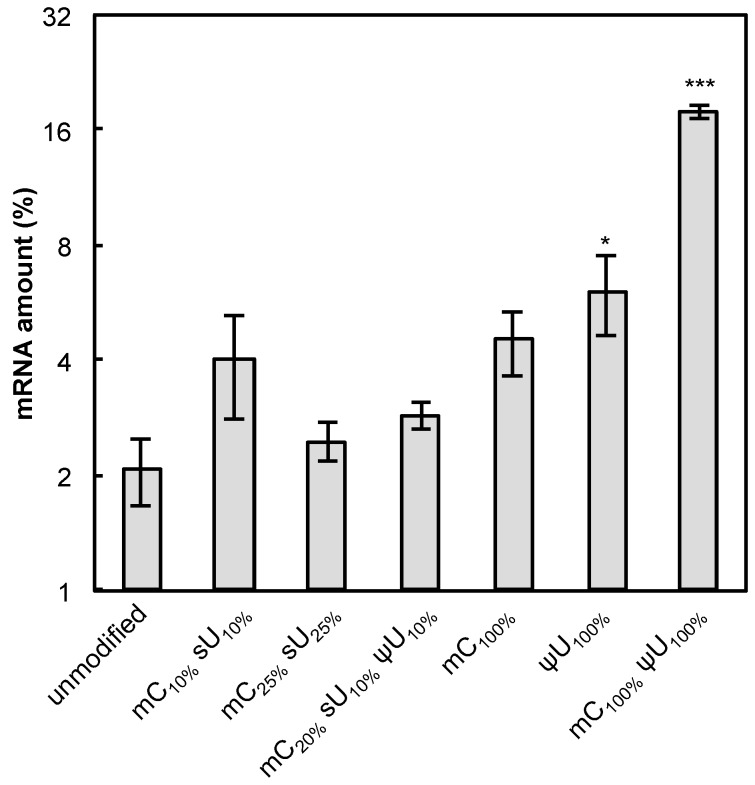
Nuclease stability of mRNA. mRNA-encoding GLuc in a naked form was incubated in 0.05% serum at 37 °C for 15 min. qRT-PCR measurement was performed to quantify the amount of mRNA that has an intact sequence between a chosen primer pair. The amount of mRNA after serum incubation was compared to that before serum incubation and the relative values are shown in the graph. Data are presented as the mean ± standard error of the mean (S.E.M.) (*N* = 4). Statistical significance was assessed by one-way analysis of variance (ANOVA) followed by Dunnett’s test. * *p* < 0.05, *** *p* < 0.001 *versus* unmodified mRNA.

Another factor that might affect protein expression is the nuclease stability of mRNA under physiological conditions. To evaluate this, the modified mRNA formulations were incubated in naked form in 0.05% serum for 15 min at 37 °C. The amount of preserved mRNA was quantified by qRT-PCR. The modified mRNA generally showed enhanced stability compared with unmodified mRNA (Figure 7). In particular, the mRNA containing ψU_100%_ or mC_100%_ ψU_100%_ exhibited significantly higher stability, corresponding to the high protein expression detected in Raw 264.7 cells or MSCs (Figure 2, Figure 3, Figure 4 and Figure 5).

## 4. Discussion

Chemical modification of mRNA is widely used as a technique to reduce the immunogenicity of mRNA. Previous studies showed that inflammatory responses after transfection of unmodified mRNA negatively affect protein production efficiency [22,23]. Similarly, for a macrophage-derived cell line capable of secreting inflammatory cytokines, the protein expression after mRNA transfection generally showed an inverse correlation with the degree of cytokine induction and a positive correlation with cell viability (Figure 2b,c and Figure S2). This suggests the inflammatory response could adversely affect cell conditions or homeostasis, resulting in reduced protein production. Certain mRNA modification could alleviate the adverse effect. However, using non-immune cell types, we found that mRNA modification did not always enhance protein expression, and the outcomes varied for the modification formulations. Several possible factors that could affect protein production efficiency were evaluated. Translational efficiency of mRNA, evaluated in a cell-free translation system, decreased for all mRNA modifications evaluated in this study (Figure 6). In contrast, evaluation of nuclease stability in serum revealed that mRNA modification generally enhanced the stability (Figure 7). Thus, mRNA modification is likely to exert both positive and negative effects on the efficiency of protein expression in transfected cells.

In addition to the effects attributed to mRNA formulations, the protein expression fluctuated among transfection reagents, cell types, and the encoded genes. Even when the same formulation of modified mRNA was used, opposite effects of increasing or decreasing the protein expression compared with unmodified mRNA were observed depending on the different reagents and cell types. These contradictory results are likely due to the complicated processes of transgene expression from mRNA uptake to protein translation in the cells. For example, it is reasonable to assume that the cell uptake speed of the mRNA or mRNA-loaded particles would be different among various cell types, and the speed might be considerably altered not only by the inflammation but also the particle size and properties [24]. When the uptake speed is low, nuclease stability of mRNA in culture medium will play a key role in increasing the protein expression. In contrast, when the speed is high, the translational efficiency from the mRNA taken up into the cells would be the more important factor to determine the final outcome of protein expression.

Because of these complicated situations, it is essential to screen the mRNA modification formulations each time to obtain sufficient protein expression. For example, mRNA with mC_100%_ or mC_100%_ ψU_100%_ showed the lowest inflammatory responses (Figure 1), but had a lower protein expression than other formulations in some cases (Figure 3, Figure 4 and Figure 5). Thus, the optimal formulation should be determined based on target cell type and transfection purpose. For example, in regenerative medicine, the formulation to minimize the immunogenicity would be preferable for preserving cell homeostasis. In contrast, for vaccination, it is acceptable to use a formulation that provides maximum protein expression, even if it induces an inflammatory response.

## 5. Conclusions

It is difficult to determine the optimal formulation for modified mRNA because, although most formulations have reduced immunogenicity, the protein expression in transfected cells varied significantly with cell type. Modifications generally decreased the translation efficiency from the mRNA but increased the nuclease stability of mRNA, suggesting that the mRNA modification is likely to exert both positive and negative effects on the efficiency of protein expression in transfected cells. Thus, it is recommended that mRNA formulation be optimized for target cell type and transfection purposes.

## Supplementary Files

Supplementary File 1
