# Supplementary material for: Screening of mRNA Chemical Modification to Maximize Protein Expression with Reduced Immunogenicity
Source: Pharmaceutics. 2015 Jul 23;7(3):137–51. doi: 10.3390/pharmaceutics7030137 (PMC4588190; doi:10.3390/pharmaceutics7030137)
Supplement: Supplementary File 1 [file pharmaceutics-07-00137-s001.pdf]

Supplementary Information

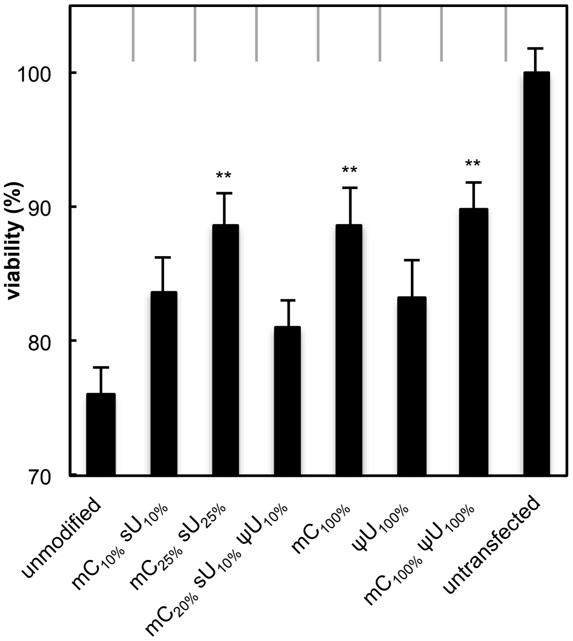

Figure S1. Cell viability after mRNA introduction to RAW264.7 cells. mRNA encoding GLuc was introduced to RAW264.7 cells using Lipofectamine™ LTX. After 4 h, cell viability was measured. Data are presented as the mean ± standard error of the mean (S.E.M.) (N = 6). Statistical significance was assessed by one-way analysis of variance (ANOVA) followed by Dunnett's test. **, $p < 0.01$ versus unmodified mRNA.

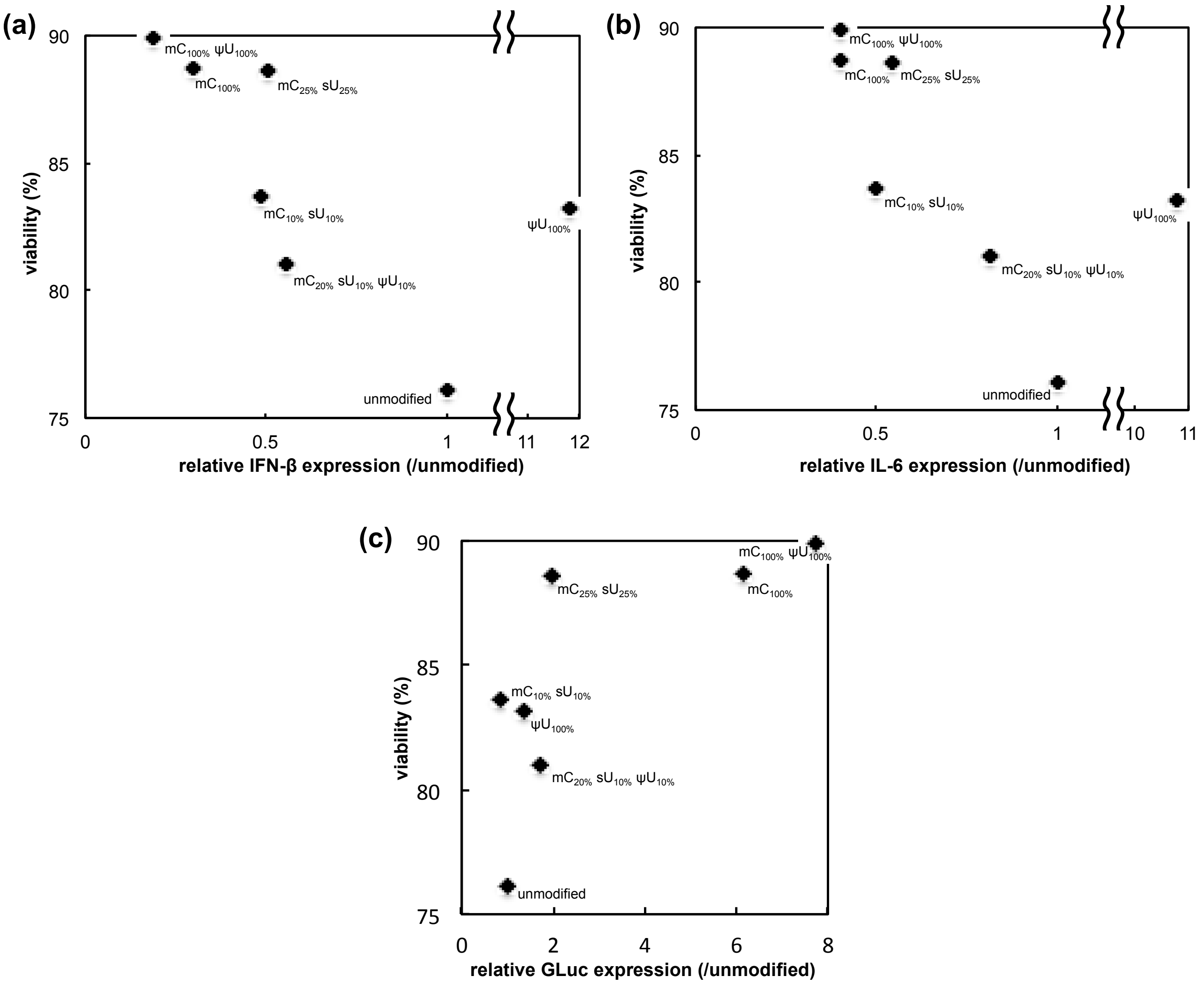

Figure S2. Correlation between cell viability, inflammatory responses, and GLuc expression. mRNA encoding GLuc was introduced to RAW264.7 cells using Lipofectamine™ LTX. After 4 h, cell viability (see Figure S1), expression of inflammatory molecules (see Figure 1), and GLuc expression (see Figure 2a) were measured. (**a**,**b**) Correlation between inflammatory responses and cell viability. (**c**) Correlation between GLuc expression and cell viability. The *x*-axis shows the expression of (**a**) interferon-β (IFN-β), (**b**) interleukin-6 (IL-6), and (**c**) GLuc. The *y*-axis showed the cell viability.

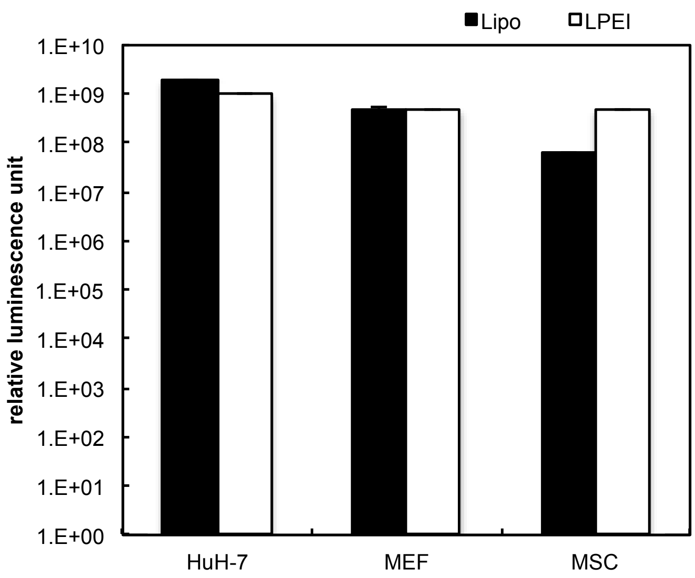

Figure S3. Comparison of GLuc expression efficiency by transfection of unmodified mRNA between two different transfection reagents. Unmodified mRNA was introduced to HuH-7 cells, MEFs and MSCs, using two types of transfection reagents, Lipofectamine™ LTX (Lipo, black bars) and linear polyethyleneimine (LPEI, white bars). The data are used in Figure 4 to calculate the "relative GLuc expression" by the modified mRNAs. Data are presented as the mean ± standard error of the mean (S.E.M.) ($N = 6$).

© 2015 by the authors; licensee MDPI, Basel, Switzerland. This article is an open access article distributed under the terms and conditions of the Creative Commons Attribution license (http://creativecommons.org/licenses/by/4.0/).
